# Warthin’s Tumor: A Diagnostic Challenge as a Metastatic Mimicker on Post-I-131 Therapy Scan

**DOI:** 10.7759/cureus.103721

**Published:** 2026-02-16

**Authors:** Fatma M Al Hajri, Naima Al Bulushi, Zakiya Al-Ajmi, Zamzam Al Bimani

**Affiliations:** 1 Nuclear Medicine, Royal Hospital, Muscat, OMN; 2 Laboratory Medicine and Pathology, Royal Hospital, Muscat, OMN

**Keywords:** false positive i-131, i-131 uptake, parotid lesion, thyroid cancer, warthin tumor

## Abstract

We present a rare case with an incidental finding of Warthin’s tumour demonstrating I-131 uptake on a post-therapy whole-body scan in a patient treated for thyroid cancer with total thyroidectomy. The unexpected radioiodine concentration in the parotid region raised initial concern for metastatic disease, illustrating how non-thyroidal lesions can mimic pathological findings in post-ablative imaging. This case highlights a well-recognised diagnostic challenge in nuclear medicine: false-positive I-131 uptake. Differentiating benign radioiodine-avid lesions from true metastases is critical to prevent incorrect staging, unnecessary interventions, and inappropriate management decisions in thyroid cancer follow-up.

## Introduction

Warthin’s tumour (papillary cystadenoma lymphomatosum) is a common benign salivary gland neoplasm, accounting for approximately 10-20% of all parotid gland tumours, and is the second most prevalent benign parotid tumour. It is typically slow growing, may be bilateral or multifocal, and is characterized histologically by oncocytic epithelium with prominent lymphoid stroma [1,2].

In nuclear medicine practice, Warthin’s tumours are well recognized for avid tracer uptake on functional imaging. Although intense fluorine-18 fluorodeoxyglucose (¹⁸F-FDG) uptake is well described, rare cases of radioiodine retention have been reported. This finding is clinically significant in patients undergoing Iodine-131 (I-131) whole-body scintigraphy for differentiated thyroid carcinoma, as it may mimic metastatic disease and result in false-positive interpretation [3].

## Case presentation

A woman in her mid-70s was referred to our institution after undergoing a total thyroidectomy for a suspicious follicular neoplasm of the Hurthle cell type.

**Figure 1:**
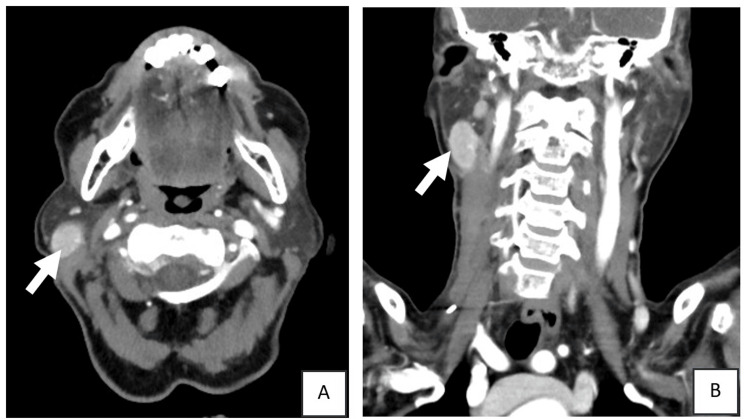
Contrast-enhanced CT images of the neck in (A) axial and (B) coronal planes demonstrate a well-circumscribed, oval-shaped, avidly enhancing soft-tissue lesion located within the right parotid gland (white arrows). The lesion shows homogeneous enhancement without central necrosis or calcification. No surrounding fat stranding or invasive features are seen. These imaging features are compatible with a benign parotid lesion.

**Figure 2:**
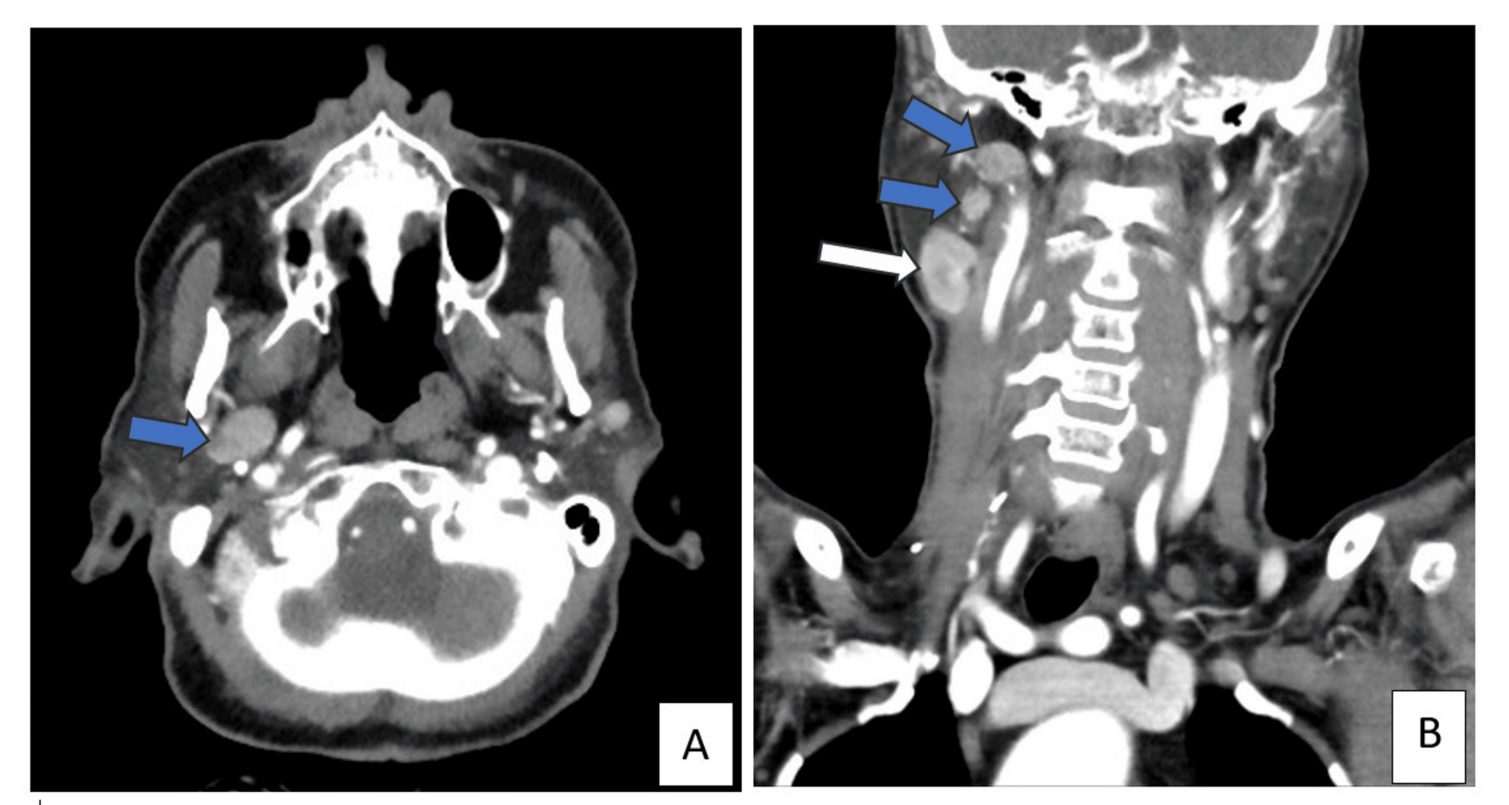
Contrastenhanced CT scan, (A) axial and (B) coronal views, of the neck at the level of parotid gland Few enhancing right cervical lymph nodes are noted (blue arrows) and a well-defined oval shaped hyper-enhancing lesion within the right parotid gland (Warthin’s tumour) (white arrow).

**Figure 3:**
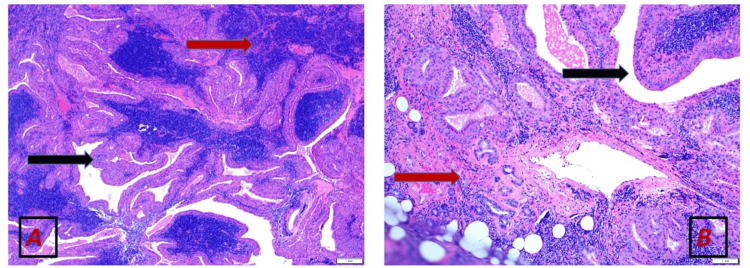
Hematoxylin and eosin stained section at (A) 5× magnification, and (B) 10× magnification (A) The lesion is composed of papillary structures lined by bi-layered oncocytic epithelium, forming cystic spaces (Black arrow). The surrounding stroma exhibits dense lymphocytic cell infiltrate (Red arrow). The morphologic features are characteristic of Warthin’s tumour of salivary glands. (B) Focal residual seromucinous glands are seen (Red arrow) in keeping with salivary gland tissue. Part of the tumour is also noted in the field (Black arrow).

**Figure 4:**
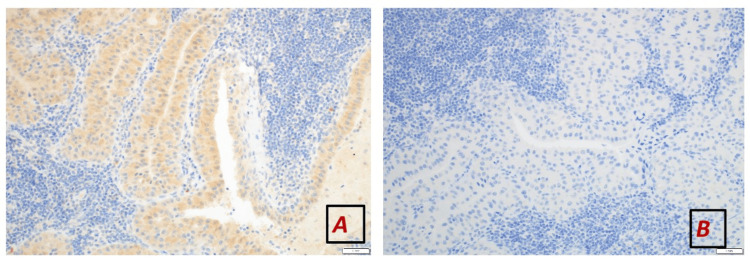
The tumour cells are negative for (A) TTF-1 (10x magnification power) and (B) thyroglobulin (20x magnification power) thyroid specific markers.

**Figure 5:**
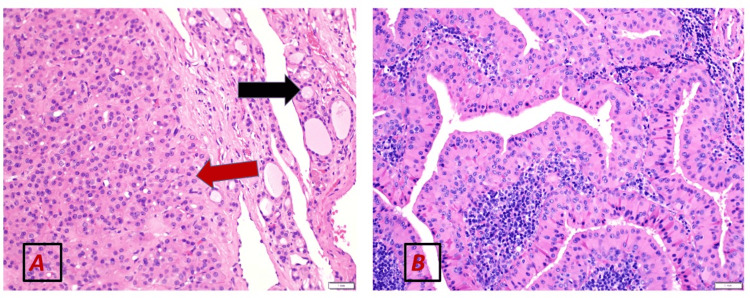
Hematoxylin and eosin stained sections (20 x magnification power) (A) Thyroid oncocytic carcinoma (Red arrow) is seen from the same patient’s thyroid lesion. Normal thyroid tissue is present (Black arrow). (B) Stained section from the Warthin’s tumour for comparison as the thyroid lesion lacks the papillary structures and the lymphoid rich stroma.

Histopathology confirmed oncocytic carcinoma with no lymph node metastasis (pT2N0, American Joint Committee on Cancer (AJCC) staging system, 8th edition [4]). A neck ultrasound detected a 3.0 x 1.8 x 1.2 cm nodule in the right parotid gland one month post-surgery. Subsequent contrast-enhanced neck CT (Figures 1, 2) showed an enhancing soft tissue lesion within the right parotid gland, and multiple other suspicious enhancing right cervical lymph nodes. A right level II cervical lymph node excisional biopsy was performed and revealed a Warthin tumour (Figures 3-5).

**Figure 6:**
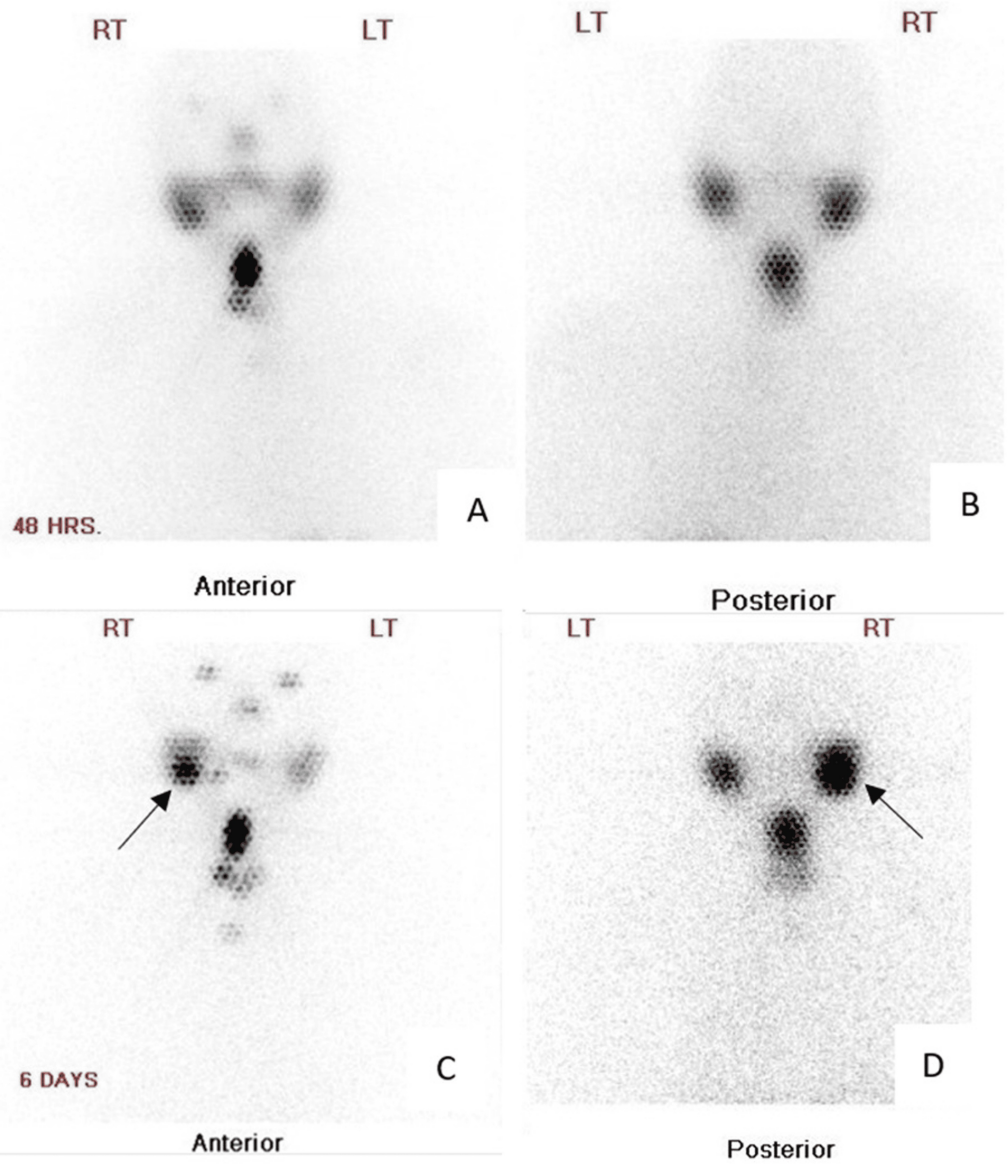
Post-radioiodine ablation therapy scan at the neck region. (A and B) Anterior and posterior projections at 48 hours after radioiodine ablation therapy scan illustrate uptake of I-131 at the surgical bed, denoting residual thyroid tissues. (C and D) Anterior and posterior projections at six days after radioiodine ablation therapy scan illustrate abnormal focal I-131 uptake within the right parotid region (black arrow).

**Figure 7:**
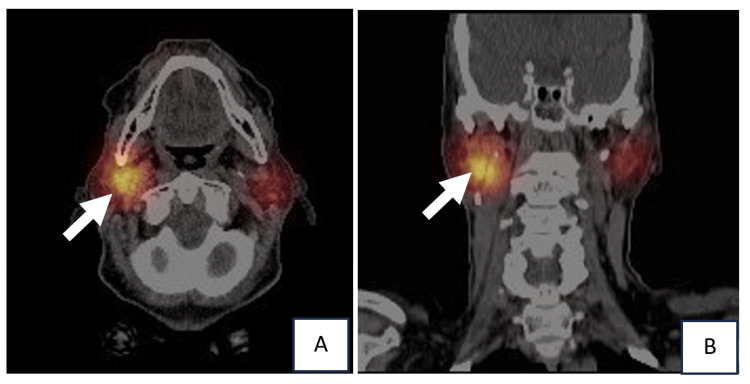
(A) Axial and (B) coronal fused SPECT/CT images demonstrate focal abnormal I-131 uptake within the right parotid gland (white arrow). SPECT: single-photon emission computed tomography; CT: computed tomography

The patient subsequently received radioiodine ablation therapy with 3.7 GBq (100 mCi) of I-131 NaI following two thyrogen (rhTSH) injections as part of thyroid cancer treatment. Delayed whole-body scans at two and six days post therapy revealed abnormal I-131 uptake in the midline neck and right parotid region (Figure 6). Single-photon emission computed tomography (SPECT)/CT correlated these to residual thyroid tissue and an intra-parotid lymph node or parotid lesion (Figure 7). The findings indicate an iodine-avid soft tissue lesion in the right parotid gland, most likely representing a Warthin’s tumour.

Additional clinical evidence included serial thyroglobulin measurements demonstrating a progressive decline (1.47, 0.73, and 0.07 µg/L at approximately one, three, and nine months post total thyroidectomy, respectively; reference <0.1 µg/L post-thyroidectomy), with consistently negative anti-thyroglobulin antibodies (Table 1), and histopathological confirmation of Warthin's tumour cells in the right cervical lymph nodes, also supports the diagnosis. No distant metastases were observed.

**Table 1:** Serial thyroglobulin and antithyroglobulin measurements following total thyroidectomy. TSH: thyroid stimulating hormone

Time from Surgery	Thyroglobulin (µg/L) (Ref: <1–2 post-thyroidectomy)	Anti-Thyroglobulin (IU/mL) (Ref: <1.3)	TSH (mIU/L) (Ref: 0.55–5.0)
~1-month post-surgery	1.470	< 1.3 (negative)	8.714 ↑
~3 months post-surgery	0.731	< 1.3 (negative)	0.176 ↓
~9 months post-surgery	0.070	< 1.3 (negative)	22.151 ↑

## Discussion

Warthin’s tumours, also known as papillary cystadenoma lymphomatosum [3], are benign salivary gland tumours that can occasionally complicate the interpretation of post-I-131 therapy scans, causing an upgrade in the staging of thyroid cancer. The uptake of I-131 by Warthin’s tumours can lead to false-positive results [5,6], which may mimic metastatic thyroid cancer, posing a diagnostic challenge and potentially leading to unnecessary aggressive management. Histopathologically, Warthin's tumour is characterised by papillary and cystic structures lined by bilayered, bland, oncocytic epithelial cells with abundant granular, eosinophilic cytoplasm, set within a lymphoid-rich stroma frequently containing reactive germinal centres [7]. Notably, both Warthin’s tumour and oncocytic (Hürthle cell) carcinoma are characterised by mitochondria-rich oncocytes, which account for their histological resemblance and contribute to diagnostic mimicry between benign salivary lesions and metastatic thyroid disease [8]. In contrast, oncocytic (Hürthle cell) carcinoma of the thyroid requires evidence of capsular and/or vascular invasion, while the Warthin-like variant of papillary thyroid carcinoma is distinguished by classical papillary thyroid carcinoma nuclear features, which are absent in Warthin's tumour. In addition, Warthin's tumour may occur as an intranodal inclusion, potentially mimicking metastatic oncocytic thyroid carcinoma, highlighting the importance of careful histopathological assessment, particularly given the limitations of fine-needle aspiration cytology [7].

The mechanism behind I-131 uptake in Warthin’s tumours is not entirely understood, but several hypotheses have been proposed. One possible explanation is the presence of sodium/iodide symporter (NIS) expression in the tumour cells. NIS is responsible for the active transport of iodide into thyroid cells, and its expression in non-thyroidal tissues, including some benign and malignant tumours, can lead to iodide uptake. In Warthin’s tumours, impaired excretory function compared to normal salivary gland tissues may contribute to the retention of radioiodine, leading to increased I-131 uptake [9].

Additionally, Warthin’s tumours are known for their cystic and lymphoid-rich nature, which can further complicate imaging interpretation. The slow exchange of water and chemical elements between the cystic components and their surrounding extracellular environment can result in the trapping of radioiodine within the cysts. This phenomenon is similar to other cystic structures, such as nasolacrimal sac, pleuropericardial, bronchogenic, thymic, breast, hepatic, renal, ovarian, epithelial, and sebaceous cysts, which are also known to show false-positive findings on radioiodine scans due to passive diffusion or partially active transport of iodide [10].

Various imaging modalities such as ultrasound, CT, MRI, Thallium-201, and ^18^F-FDG PET scans play a role in evaluating these tumours. Each modality offers unique advantages, from anatomical detail to metabolic activity assessment. While imaging provides critical insights, these findings must always be considered with biopsy results and clinical context to avoid misdiagnosis. The totality of information is crucial in making an accurate diagnosis, as reliance on imaging alone can lead to overestimating disease severity.

Moreover, Warthin’s tumours have been shown to exhibit increased uptake with other radiotracers, namely Thallium-201 and ^18^F-FDG PET [11]. This increased uptake is likely related to the metabolic activity of the tumour cells and the presence of lymphoid tissue, which can exhibit avidity for various radiotracers.

Compared to previously reported cases of I-131 uptake in Warthin’s tumour [3,8,12], this case highlights that enhancing cervical lymph nodes along with an enhancing intra-parotid lesion should not always be presumed to represent metastases from thyroid cancer, as these findings may also demonstrate uptake on post-I-131 therapy scans.

Recognition of Warthin’s tumours and other causes of I-131 uptake in the salivary glands, including saliva retention in the ducts or glands, salivary gland inflammation (sialadenitis), and oncocytomas [13], as potential sources of false-positive I-131 uptake, is crucial for accurate interpretation of post-radioiodine therapy whole-body scintigraphy.

Advances in imaging techniques, such as using reconstruction software with attenuation correction, allow for the quantitative characterisation of lesions. This can aid in differentiating benign from malignant lesions, potentially leading to more accurate diagnoses and reducing the likelihood of unnecessary treatments [12].

Management

The preferred treatment for Warthin’s tumour is surgical excision, which is relatively straightforward due to the tumour’s superficial location. The probability of malignant transformation in Warthin’s tumours is low, approximately 0.3%, classifying it as a definitively benign neoplasm of the salivary glands [14]. For this reason, enucleation is often considered an appropriate treatment approach [15]. In some cases, surveillance may also be a reasonable option, particularly in asymptomatic patients or those for whom surgery presents an increased risk. These tumours’ slow-growing and benign nature allows for periodic monitoring through imaging, thus avoiding the risks associated with surgical intervention. While Warthin’s tumours can occasionally involve cervical lymph nodes, this is not considered a true metastasis. The management remains primarily surgical, with complete excision of the tumour and involved lymph nodes, followed by routine surveillance [16]. Management decisions should be individualised, considering the patient’s clinical situation, preferences, and potential risks associated with intervention versus observation.

## Conclusions

This case highlights the diagnostic complexity of such findings in thyroid cancer patients. Specifically, it demonstrates that enhancing cervical lymph nodes accompanied by an enhancing intra-parotid lesion should not automatically be interpreted as metastatic thyroid cancer, as these findings may represent benign entities such as Warthin’s tumour, which can also exhibit uptake on post-I-131 therapy scans. Clinically, when isolated parotid uptake is encountered on post-therapy I-131 scans, correlation with thyroglobulin levels and consideration of targeted salivary or anatomical imaging should be undertaken before escalation to aggressive surgical management. Recognition of this mimic is essential to prevent unnecessary upstaging and avoid overtreatment, including radical neck dissection.
